# Ultrasound screening for cholangiocarcinoma could detect premalignant lesions and early-stage diseases with survival benefits: a population-based prospective study of 4,225 subjects in an endemic area

**DOI:** 10.1186/s12885-016-2390-2

**Published:** 2016-06-02

**Authors:** Prakongboon Sungkasubun, Surachate Siripongsakun, Kunlayanee Akkarachinorate, Sirachat Vidhyarkorn, Akeanong Worakitsitisatorn, Thaniya Sricharunrat, Sutida Singharuksa, Rawisak Chanwat, Chairat Bunchaliew, Sirima Charoenphattharaphesat, Ruechuta Molek, Maneenop Yimyaem, Gaidganok Sornsamdang, Kamonwan Soonklang, Kasiruck Wittayasak, Chirayu U. Auewarakul, Chulabhorn Mahidol

**Affiliations:** Chulabhorn Hospital, 54 Kamphaeng Phet 6 Road, Laksi, Bangkok, 10210, Thailand; Ban Luang Hospital, 191 Pa Kha Luang, Ban Luang, Nan, 55190 Thailand; Faculty of Medicine Siriraj Hospital, Mahidol University, 2 Wanglang Road, Bangkoknoi, Bangkok, 10700 Thailand; National Cancer Institute of Thailand, 268/1 Rama VI Road, Ratchathewi, Bangkok, 10400 Thailand; Chulabhorn Research Institute, 54 Kamphaeng Phet 6 Road, Laksi, Bangkok, 10210 Thailand

**Keywords:** Cholangiocarcinoma, Premalignant lesions, Cancer screening, Early detection, Ultrasonography, Tumor markers

## Abstract

**Background:**

Thailand has a high incidence of cholangiocarcinoma (CCA), particularly in the north and northeastern regions. Most CCA patients come at a late, unresectable stage and presently no optimal screening test for CCA has been established. We determined the prevalence of CCA in a remote northern village and explored if screening could lead to early detection and survival benefits.

**Methods:**

A 5-year population-based study was started in October, 2011 for consented Thai individuals, aged 30–60 years. The screening program comprised blood testing, stool examination and serial ultrasonography every 6 months.

**Results:**

During the first 3 years, 4,225 eligible individuals were enrolled. CCA was detected in 32 patients, with a mean age of 51.9 years (41–62 years), and 21/32 cases were at a curative resectable stage. The prevalence rate of CCA was 165.7 per 100,000 and one- and two-year incidence rate was 236.7/100,000 and 520.7/100,000, respectively. One- and 2-year overall survival rates of CCA patients were 90.9 and 61.5 %, respectively. Prognosis was better in resectable cases with 100 % 1-year and 77.8 % 2-year survival rates. Interestingly, premalignant pathological lesions (stage 0) were identified in 11 cases with 100 % 3-year survival rate. Serum biomarkers and alkaline phosphatase were not sufficient to detect early-stage disease. In 22 patients, stool samples were positive for *Opistorchis viverrini*, based on polymerase chain reaction.

**Conclusion:**

Detection of premalignant lesions and early-stage resectable CCA by ultrasonography resulted in improved clinical outcome. Ultrasonography should be offered as a first screening tool for CCA in an endemic area until other useful biological markers become available.

## Background

Cholangiocarcinoma is a tumor of the biliary tract, presumably of cholangiocytic origin, with a rising global incidence [1–6]. Several known risk factors exist, linking chronic biliary inflammation to the pathogenesis of cholangiocarcinoma [7, 8]. Late presentation to hospital, with a median survival of months, is noted in most patients in developing countries whereby cholangiocarcinoma is most prevalent. Moreover, the pathological or cytological diagnosis of cholangiocarcinoma is not always accessible despite indications from imaging studies and clinical condition [7–10]. Surgical resection is the current therapy of choice for every type of cholangiocarcinoma [8, 11, 12]. Resection offers the best opportunity for long-term survival. Nevertheless, in the minority of patients and in those with large node-positive or multifocal intrahepatic cholangiocarcinoma, resection seems to provide little benefit [10, 12]. Overall, the 5-year survival in cholangiocarcinoma cases is poor, with 60 % to >90 % recurrence rates [7, 9, 13].

Cholangiocarcinoma is relatively rare in most western countries, but high incidence rates have been reported in Eastern Asia, especially in Thailand [5, 14–16]. The etiology of this cancer appears to be mostly due to specific infectious agents [17, 18]. In 2009, infections with liver flukes, *Clonorchis sinensis* or *Opistorchis viverrini*, were both classified as carcinogenic to humans by the International Agency for Research on Cancer for cholangiocarcinoma [14, 18]. With the current systematic tumor registration in Thailand, new high-incidence areas have been identified in North and Northeastern Thailand [10, 17, 19]. Ban Luang is a district in the western part of Nan Province in Northern Thailand and is divided into 4 sub-districts. Based on a previous study, the incidence of liver cancer in Ban Luang was 138.8 per 100,000 persons, which is higher than that of other regions of the world or even in Khon Kaen Province, which is previously reported as an endemic area for cholangiocarcinoma [17, 20, 21].

The present study aimed to ascertain the prevalence and incidence of cholangiocarcinoma, to identify predisposing factors, and to explore whether screening could lead to early treatment and reduction of morbidity and mortality rates of cholangiocarcinoma patients.

## Methods

### Study design and population

A population-based, prospective cohort study for cholangiocarcinoma screening included liver ultrasonography, stool examination for parasites, complete blood count (CBC), liver function tests (LFT) including alkaline phosphatase (ALP), and measurements of hepatitis B surfaceantigen, hepatitis B core antibody, and serum carcinoembryonic antigen (CEA), carbohydrate antigen (CA)19-9, and α-fetoprotein (AFP) every 6 months from October 2011 to September 2016. This study was approved by the Ethics Committee for Human Research of Chulabhorn Research Institute, Bangkok, Thailand (Certificate no. 29/2554). Written informed consents were obtained from all the study participants.

Targeted subjects were all indigenous residents of Ban Luang District, aged 30–60 years, who were not pregnant, or breast feeding, or diagnosed with or under treatment for any type of cancer. Of 6,327 targeted subjects based on a district census registration, 4,337 consented to the study and were recruited by village health volunteers with the cooperation of Ban Luang Hospital. Natural history and prevalence and incidence rates of cholangiocarcinoma were investigated, along with an analysis of associated risk factors and a comparison of results between liver ultrasonography and laboratory testing. People with liver lesions suspected of liver cancer, such as isolated mass lesions, masses associated with bile duct dilatation, or isolated bile duct dilatation without mass lesions were referred for further imaging studies, including computed tomography (CT), magnetic resonance imaging (MRI) or magnetic resonance cholangiopancreatography (MRCP) at Chulabhorn Hospital. All cholangiocarcinoma treatments, that is, surgery, chemotherapy or radiotherapy, were performed at Chulabhorn Hospital.

### Laboratory and ultrasonography studies

LFT and tumor markers were performed by Cobas 6000 (c501 and e601) of Roche Diagnostics (Thailand) and liver ultrasonography was performed using *Logiq C2* ultrasound system (*GE* Healthcare) and Aplio 300 and 500 ultrasound system (Toshiba). Recommended diagnostic cut-off value for CA19-9, CEA and alkaline phosphatase (ALP) was >37 U/mL, ≥4.7 ng/mL, and >100 IU, respectively. Liver ultrasonography was performed by a team of radiologists from Chulabhorn Hospital and Nan Hospital. Criteria for further CT, MRI, and/or MRCP investigations included nodule/mass lesion, nodule/mass with bile duct dilatation, and focal bile duct dilatation. In case of diffuse bile duct dilatation without other associated abnormality, MRCP was performed to exclude small biliary intraductal lesions by using a peripheral bile duct diameter of ≥ 3 mm. Patients who were diagnosed as having suspicious/definite malignant lesions by CT, MRI, and MRCP were subsequently reviewed by a multi- disciplinary team for further treatment planning. All cancer specimens were pathologically diagnosed at Chulabhorn Hospital with routine hematoxylin and eosin (H&E) staining and immunohistochemistry was additionally performed if necessary.

### Statistical analysis

Demographic data were reported as mean and standard deviations for all continuous variables and as proportions and absolute counts for discrete variables. The Mann-Whitney *U* test was used to compare continuous variables, whereas Pearson *χ*^2^ and Fisher’s exact tests were used to compare discrete variables. A two-tailed *P* < 0.05 was considered to be significant to verify the assumptions for all statistical tests. Prevalence was calculated from cholangiocarcinoma patients detected by initial screening ultrasonography. Incidence was calculated from new cases detected by subsequent ultrasonographic studies. Disease-free survival (DFS) was defined as the length of time that the patient survived without any signs or symptoms, after primary treatment for cholangiocarcinoma was completed. Progression-free survival (PFS) was the length of time during and after treatment of cholangiocarcinoma that the patients lived with the disease, without deterioration or progression. Overall survival (OS) was the length of time that the patients were still alive, starting from the date of diagnosis or start of cholangiocarcinoma treatment.

## Results

### Demographic data of the cohort and prevalence and incidence of cholangiocarcinoma

Between October 2011 and April 2014, abdominal ultrasonography was completed in 4,225 participants (1,919 males and 2,306 females) from 4,337 recruited participants. Cholangiocarcinoma was detected in 32 patients, with a mean age of 51.9 years (41–62 years), comprising 18 men (56.3 %) and 14 women (43.7 %). Tables 1 and 2 shows a comparison between cholangiocarcinoma patients and non-cholangiocarcinoma population. There was no significant difference between cholangiocarcinoma patients and non-cholangiocarcinoma population regarding gender, smoking, history of parasitic infection and treatment, and raw freshwater animal consumption (*P* > 0.05). The mean age of cholangiocarcinoma patients was 51.9 years and that of non-cholangiocarcinoma cases was significantly lower at 45.7 years and alcohol consumption was significantly different between the 2 groups. History of unclassified liver cancer or cholangiocarcinoma in first-degree relatives was significantly higher in cholangiocarcinoma patients (33.3 %) than in the non-cholangiocarcinoma group (17.1 %).

**Table 1:** Demographic data of cholangiocarcinoma patients and non-cholangiocarcinoma population

Demographic data	Total	Cholangiocarcinoma patients (*n* = 32)	Non- cholangiocarcinoma population (*n* = 4,193)	*P* value
Gender	4,225	32	4,193	0.334^a^
Male	1,919	18	1,901	
	(45.4)	(56.3)	(45.3)	
Female	2,306	14	2,292	
	(54.6)	(43.7)	(54.7)	
Age (year)	45.71	51.91	45.68	<0.001^c^
History of smoking	4,217	27	4,190	0.092^a^
Never	2,756	14	2,742	
	(65.4)	(51.9)	(65.4)	
Ex-smoker	718	9	709	
	(17.0)	(33.3)	(16.9)	
Active smoker	743	4	739	
	(17.6)	(14.8)	(17.7)	
Alcoholic consumption	4,217	27	4,190	<0.001^b^
Never	1,315	9	1,306	
	(31.2)	(33.4)	(31.2)	
Ex-consumption	492	10	482	
	(11.7)	(37.0)	(11.5)	
Light consumption (< once a month)	1,360	4	1,356	
	(32.2)	(14.8)	(32.3)	
Moderate to heavy consumption	1,050	4	1,046	
	(24.9)	(14.8)	(25.0)	
Any liver cancers or cholangiocarcinoma in first-degree relatives	4,202	27	4,175	0.037^a^
Yes	722	9	713	
	(17.2)	(33.3)	(17.1)	
No	3,480	18	3,462	
	(82.8)	(66.7)	(82.9)	
Diagnosis of liver fluke	4,153	27	4,126	0.366^a^
Ever	753	7	746	
	(18.1)	(25.9)	(18.1)	
Never	3,293	19	3,274	
	(79.3)	(70.4)	(79.3)	
Unknown	107	1	106	
	(2.6)	(3.7)	(2.6)	

^a^ Fisher’s exact test

^b^ Pearson’s *χ* ^2^ test

^c^ Mann–Whitney *U* test

**Table 2:** Demographic data of cholangiocarcinoma patients and non-cholangiocarcinoma population

Demographic data	Total	Cholangiocarcinoma patients (32 cases)	Non- cholangiocarcinoma population (4,193 cases)	*P* value
Any treatment for liver flukes from physicians/public health personnel	4,129	27	4,102	0.233^b^
Ever	1,441	7	1,434	
	(34.9)	(25.9)	(35.0)	
Never	2,576	18	2,558	
	(62.4)	(66.7)	(62.3)	
Unknown	112	2	110	
	(2.7)	(7.4)	(2.7)	
Raw freshwater fish/shrimp/snail consumption	4,218	27	4,191	0.568^a^
Yes	526	2	524	
	(12.5)	(7.4)	(12.5)	
No	3,692	25	3,667	
	(87.5)	(92.6)	(87.5)	

^a^ Fisher’s exact test

^b^ Pearson’s *χ* ^2^ test

^c^ Mann-Whitney *U* test

Initial screening revealed 7 asymptomatic cases of cholangiocarcinoma among 4,225 participants. The prevalence rate of cholangiocarcinoma in the Ban Luang population aged 30–60 years was 165.7 per 100,000. We subsequently detected 6, 4, 5, 7 and 3 cholangiocarcinomas from each 6-month follow-up period. The 1- and 2-year incidence rates were 236.7/100,000 (10/4,225) and 520.7/100,000(22/4,225), respectively.

### Ultrasound findings, stages and resectability of cholangiocarcinoma patients

Of 32 cholangiocarcinoma patients, 10 showed masses associated with bile duct dilatation, 9 showed isolated mass lesions, 11 showed isolated bile duct dilatation, and the other 2 cases showed questionable liver masses with ultrasonography. Twenty-one cases were resectable and 11 unresectable. The most common type of cholangiocarcinoma was intrahepatic (21/32, 65.6 %). Hilar type was found in 6 cases (18.8 %) and extrahepatic type in 5 cases (15.6 %). Based on AJCC Cancer Staging Manual, Seventh edition (2010) [22], there were 5, 10, 2, 2, 8 and 5 patients in stage I, II, IIIa, IIIb, IVa and IVb cholangiocarcinoma, respectively. In all stage I patients, resections were performed. In stage II disease, 9 patients were resected and 1 patient was medically inoperable. In stage IIIa, one patient was resected and the other was unresectable. Similarly, in stage IIIb, one patient was resected and the other was unresectable. In stage IVa, 5 patients had lymph node metastasis but still resectable lesions and 3 patients were unresectable. All patients in stage IVb were unresectable due to M1 disease. Additionally, we found 11 patients with premalignant lesions (or stage 0) (Table 3). With regards to false positive ultrasonography, we had 3 cases whose surgical specimen revealed no malignancy despite suspicious CT and MRI results. The pathological reports were chronic cholangitis with cirrhosis, adenoma with periductal fibrosis and calcified fibrotic cyst.

**Table 3:** Pathological data of patients with premalignant lesions

No.	Sex	Age	Pathology
1	M	45	Intraductal cholangiocarcinoma
2	M	60	Intraductal papillary biliary neoplasm
3	F	53	Biliary epithelial neoplasm
4	F	51	Low grade dysplastic epithelium
5	F	52	Intraductal papillary biliary neoplasm
6	M	41	High grade dysplastic epithelium
7	F	59	Hyperplasia + dysplastic epithelium
8	F	59	Intraductal papillary biliary neoplasm
9	F	53	Atypical bile duct epithelium
10	F	56	Intraductal papillary biliary neoplasm
11	M	51	Intraductal papillary biliary neoplasm

### Survival rates of patients with cholangiocarcinoma and premalignant lesions

Over a follow-up period, 1- and 2-year survival rates were 90.9 and 61.5 %, respectively, for CCA cases (Table 4). In resectable cases, 1- and 2-year survival rates were 100 % (16/16) and 77.8 % (7/9). One- and two-year survival rates were lower in unresectable cases; 66.7 % (4/6) and 25 % (1/4), respectively. In resectable cases, 1- and 2-year DFS and recurrent free survival rates were 75 % (12/16) and 44.4 % (4/9), respectively. All patients with premalignant lesions had excellent outcomes after surgery (100 % OS and 100 % 2-year DFS).

**Table 4:** Survival outcomes of patients with premalignant lesions and cholangiocarcinoma

Diagnosis	Patients with Premalignant lesions		Cholangiocarcinoma patients			
			Resectable		Unresectable	
	1-year	2-year	1-year	2-year	1-year	2-year
OS	100 %	100 %	75 %	44.4 %	33.3 %	0 %
DFS/PFS	100 %	100 %	100 %	77.8 %	66.7 %	25 %

Abbreviations: *OS* overall survival, *PFS* progression free survival, *DFS* disease free survival

### Values of LFT, tumor markers and stool examination for parasites

Serum biomarkers, CA19-9, CEA or ALP were analyzed among cases with and without cholangiocarcinoma as shown in Table 5. Sensitivity of CA19-9, CEA and ALP were 18.75, 34.38 and 50.00 %, respectively. When these tumor makers were combined, the sensitivity was still low (68.75 %). Stool examination was performed in 3,663 individuals (86.67 %). There were 7 types of parasites in 824 cases (22.50 %). *O. viverrini*-like eggs were found in 710 cases (19.38 %), *Taenia* eggs in 56 cases (6.17 %), *Sarcocystis* spp*.* eggs in 41 cases (4.42 %), *Strongyloides stercoralis* eggs in 17 cases (1.85 %), hookworm eggs in 45 cases (1.23 %), *Trichuris trichiura* eggs in 5 cases (0.14 %), and *Enterobius vermicularis* eggs in 2 cases (0.05 %). In 22 of 32 cholagiocarcinoma patients, stool samples were positive for *Opistorchis viverrini*, based on polymerase chain reaction*.*

**Table 5:** Tumor marker analysis in the cases with and without cholangiocarcinoma

Predictor	Cholangiocarcinoma		Total	Sensitivity	Specificity	PPV	NPV
	Positive	Negative					
Positive CA19-9	6	59	65	18.75	98.63	9.23	99.39
Negative CA19-9	26	4245	4271				
Total	32	4304	4336				
Positive CEA	11	749	760	34.38	82.60	1.45	99.41
Negative CEA	21	3555	3576				
Total	32	4304	4336				
Positive ALP	15	641	656	50.00	83.54	2.29	99.54
Negative ALP	15	3254	3269				
Total	30	3895	3925				
Positive CA19-9 or CEA or ALP	22	1252	1274	68.75	70.91	1.73	99.67
Negative CA19-9 or CEA or ALP	10	3052	3062				
Total	32	4304	4336				

Abbreviations: *CA19-9* carbohydrate antigen 19–9, *CEA* carcinoembryonic antigen, *ALP* alkaline phosphatase, *NPV* negative predictive value, *PPV* positive predictive value

## Discussion

Cholangiocarcinoma is a silent malignancy with a significantly high morbidity and mortality. Patients usually present at a late stage, rendering curative measures impossible to be performed to prolong lives [9, 13]. Although the annual incidence rate of cholangiocarcinoma in the western countries is low at 1–2 cases per 100,000, many studies have documented a steady increase in the incidence of intrahepatic cholangiocarcinoma over the past few decades; increases have been seen in North America, Europe, Asia, and Australia [2, 4, 6]. Our study confirmed a high prevalence and high incidence rate of cholangiocarcinoma in Thailand, particularly in the northern region of the country with a prevalence rate of 165.7 per 100,000 and 1- and 2-year incidence rates of 236.7/100,000 and 520.7/100,000, respectively [10, 17].

As a screening program for cholangiocarcinoma is currently not established worldwide, this study was intended to explore if abdominal ultrasonography or tumor markers could be of benefit to detect early-stage cases for curative intent in an endemic area. By performing successive ultrasonography every 6 months, we managed to identify 32 cholangiocarcinoma cases from 1 to 6 sessions over a 3-year period and 65 % of our patients were diagnosed with early-stage and operable tumors which was markedly different from all the hospital-based data previously reported in Thailand whereby most cholangiocarcinoma cases presented at a late stage. A recent study from the National Cancer Institute of Thailand showed that there were 2.17, 12.69, 24.77, and 57.89 %, respectively, of stage I, II, III, and IV of newly diagnosed cholangiocarcinoma cases diagnosed at their institute in 2013 [23]. Nevertheless, the most common tumor location in this study was intrahepatic which was similar to other published hospital-based studies [7, 12, 24].

Over a follow-up period of 3 years, 1- and 2-year survival rates of the affected cases were high (>60 %), particularly in resectable cases (100 and 78 %, respectively) as expected, and were much better than those reported from previous studies in Thailand and Malaysia with advanced and inoperable cases [10, 11, 25]. Outcome data from a study in Khon Kaen of 411 intrahepatic cholangiocarcinoma patients revealed that only 138 cases were resectable and the mean survival time was 1,039 days in tumor stage III, 773 days in stage IVa, and 382 days in stage IVb [10]. Rare long-term survival with a one-year survival of just 15 % was reported in perihilar cholangiocarcinoma cases [11]. Another long term follow-up data from Malaysia in 69 cholangiocarcinoma patients showed that the overall median survival was 4 months with the median survival of 16 months in R0 resected patients [25]. Overall 1-, 2- and 3-year survival rates were 67, 17 and 17 %, respectively. In one study from the US, 53.8 % of newly diagnosed intrahepatic cholangiocarcinoma patients were not candidates for resection [12]. In resectable groups, the median disease-specific survival was 36 months and recurrence was observed in most of the patients (62.2 %). In unresectable groups, the median survival varied depending on modalities of treatments, i.e., 22 months and 9 months in patients receiving regional chemotherapy as part of treatment and systemic chemotherapy alone, respectively. Although our outcomes could not be directly compared because other reports were from hospital-based studies, it is evident that screening ultrasonography could identify patients at an early and asymptomatic stage leading to better treatment outcomes.

Interestingly, our study identified 11 patients with premalignant lesions (stage 0) such as biliary intraepithelial neoplasia, intraductal papillary biliary neoplasm or dysplastic epithelium [26]. Our pathological results were achievable because we detected patients at the earliest stage whereas most studies of cholangiocarcinoma did not have pathological tissue due to the inoperative nature of the late-stage cases. These premalignant lesions represent cases whereby abnormal cells were found in the innermost layer of the tissue lining of the extrahepatic bile duct. All of them did better than the early-stage cholangiocarcinoma cases with 100 % DFS and are still alive at 3-year follow-ups. Therefore, patients detected at a premalignant stage appeared to benefit the most from screening ultrasonography and potentially are cured.

With regards to demographic data, the population in Ban Luang is socioeconomically comparable to rural northern and northeastern population of Thailand with similar ethnic backgrounds [27]. The villagers’ major occupation was farming with an average income per month of 1,000–4,999 THB (about 30–145 USD). The mean age of cholangiocarcinoma cases of 51.9 years old in this study was consistent with the average age of cholangiocarcinoma cases reported from the National Cancer Registry or hospital-based studies [28] but was much lower than most western data whereby most patients are in their 60s or 70s [2, 4, 13]. During our 3-year study period, no cancer patients under the age of 40 years old were identified. Hence, age could be another factor in disease development and cholangiocarcinoma screening in individuals younger than 40 years old may not be of value or necessary in an endemic area. Similar to some previous data, the prevalence of cholangiocarcinoma was slightly higher in males as compared to females [29, 30]. Other significant demographic data was a history of alcohol drinking and a family history of liver cancer in the cholangiocarcinoma cases as compared to non-cancer cohort, which could be important for the development of cholangiocarcinoma. In addition, we found a strong family history of cholangiocarcinoma in some patients. Cholangiocarcinoma patients had many first-degree relatives with liver cancer although most of them were diagnosed only by clinical suspicion, for example, obstructive jaundice with abdominal mass, mostly without histological confirmation.

Although risk factors for the development of cholangiocarcinoma in the western countries are primary sclerosing cholangitis, an inflammatory disease of the biliary tract, and ulcerative colitis, certain parasitic liver diseases are notable risk factors in Asia. Colonization with the liver flukes *O. viverrini* (in Thailand, Laos and Vietnam) [31–33] or *Clonorchis sinensis* (in China, Taiwan, Eastern Russia, Korea, and Vietnam) is associated with the development of cholangiocarcinoma [34]. In this study, we found that about 18–25 % of our villagers had a history of liver flukes and 25–35 % of them had been treated with drugs to eradicate the parasites but no significant difference was observed between the cancer cases and the non-cancer cases. There are many potential problems with detecting *O. viverrini* eggs by microscopic examination. For example, the egg features are similar to those of lecithodendrid and heterophyid parasites, therefore, the examination requires a high level of skill, and the methods are time consuming. To overcome these constraints, with the cooperation of the Department of Helminthology, Faculty of Tropical Medicine, Mahidol University, stool internal transcribed spacer (ITS)-PCR, which is a more sensitive and specific method, was performed in 22 cholangiocarcinoma patients during the first 2 years of the study [35]. All patients had positive results from stool PCR for *O. viverrini*, suggesting that *O. viverrini* continues to be an important contribution factor to the development of cholangiocarcinoma in Thailand.

Although we could detect early-stage disease and the majority of patients were able to receive curative surgery, the recurrence rate was still not as low as expected, necessitating our attempts to identify if tumor markers could be of better benefit than ultrasonography for early screening. Our study confirmed that currently available tumor markers have inadequate sensitivity and specificity for cholangiocarcinoma screening [36, 37]. These markers, such as CEA, CA 19–9, ALP, were not sensitive enough to detect early cholangiocarcinoma cases in our study cohort. Other new biological markers may be needed to identify patients at the earliest stage that will potentially lead to curative success [35, 38, 39]. Nevertheless, with the excellent outcome of our patients with premalignant lesions, we suggest that ultrasonography should be a first screening tool for cholangiocarcinomain an endemic area until other useful biological markers are discovered or become available. Studies are ongoing to identify new genomic and proteomic markers for cholangiocarcinoma cases in Thailand [40]. New therapeutic advances are also needed to improve the disease-free survival of patients undergoing surgical resection [41, 42].

## Conclusions

This study represents the first large population-based screening program ever performed in an endemic area of cholangiocarcinoma by serial ultrasonography coupled with laboratory tests. The high prevalence and incidence rates of cholangiocarcinoma in Northern Thailand were evident. Current tumor markers and stool examination for parasites are not of benefit for cholangiocarcinoma screening in an endemic area. Ultrasonography should be offered as a first screening tool for cholangiocarcinoma in individuals aged ≥40 in an endemic area. Patients with premalignant lesions achieved the most benefit from screening ultrasonography and are potentially cured. Future studies are needed to identify new biological markers that will capture cancer cells at the earliest stage of development.

## Abbreviations

AFP, α-fetoprotein; ALP, alkaline phosphatase; CA 19–9, carbohydrate antigen 19–9; CBC, complete blood count; CCA, cholangiocarcinoma; CEA, carcinoembryonic antigen; CT, computed tomography; DFS, disease-free survival; H&E, hematoxylin and eosin; ITS, internal transcribed spacer; IU, international unit; LFT, liver function tests; mL, millilitre; mm, millimeter; MRCP, magnetic resonance cholangiopancreatography; MRI, magnetic resonance imaging; ng, nanogram; NPV, negative predictive value; *O. viverrini*, *Opistorchis viverrini;* OS, overall survival; PCR, polymerase chain reaction; PFS, progression-free survival; PPV, positive predictive value; U, unit.
